# In Situ Growth of All‐Inorganic Perovskite Single Crystal Arrays on Electron Transport Layer

**DOI:** 10.1002/advs.201902767

**Published:** 2020-04-22

**Authors:** Xiaobing Tang, Wei Chen, Dan Wu, Aijing Gao, Gaomin Li, Jiayun Sun, Kangyuan Yi, Zhaojin Wang, Guotao Pang, Hongcheng Yang, Renjun Guo, Haochen Liu, Huaying Zhong, Mingyuan Huang, Rui Chen, Peter Müller‐Buschbaum, Xiao Wei Sun, Kai Wang

**Affiliations:** ^1^ Guangdong‐Hong Kong‐Macao Joint Laboratory for Photonic‐Thermal‐Electrical Energy Materials and Devices Shenzhen Key Laboratory for Advanced Quantum Dot Displays and Lighting Department of Electrical and Electronic Engineering Southern University of Science and Technology 1088 Xueyuan Blvd. Shenzhen 518055 China; ^2^ Physik‐Department Lehrstuhl für Funktionelle Materialien Technische Universität München James‐Franck‐Straße 1, 85748 Garching Germany; ^3^ Academy for Advanced Interdisciplinary Studies Southern University of Science and Technology 1088 Xueyuan Blvd. Shenzhen 518055 China; ^4^ Department of Physics Southern University of Science and Technology 1088 Xueyuan Blvd. Shenzhen 518055 China; ^5^ Shenzhen Planck Innovation Technology Co., Ltd. Shenzhen 518129 China; ^6^ Heinz Maier‐Leibnitz Zentrum (MLZ) Technische Universität München Lichtenbergstrasse. 1, 85748 Garching Germany

**Keywords:** CsPbBr_3_ single crystals, perovskites, cubic ZnO, electron‐transport layers, optoelectronic devices

## Abstract

Directly growing perovskite single crystals on charge carrier transport layers will unravel a promising route for the development of emerging optoelectronic devices. Herein, in situ growth of high‐quality all‐inorganic perovskite (CsPbBr_3_) single crystal arrays (PeSCAs) on cubic zinc oxide (c‐ZnO) is reported, which is used as an inorganic electron transport layer in optoelectronic devices, via a facile spin‐coating method. The PeSCAs consist of rectangular thin microplatelets of 6–10 µm in length and 2–3 µm in width. The deposited c‐ZnO enables the formation of phase‐pure and highly crystallized cubic perovskites via an epitaxial lattice coherence of (100)_CsPbBr3_∥(100)_c‐ZnO_, which is further confirmed by grazing incidence wide‐angle X‐ray scattering. The PeSCAs demonstrate a significant structural stability of 26 days with a 9 days excellent photoluminescence stability in ambient environment, which is much superior to the perovskite nanocrystals (PeNCs). The high crystallinity of the PeSCAs allows for a lower density of trap states, longer carrier lifetimes, and narrower energetic disorder for excitons, which leads to a faster diffusion rate than PeNCs. These results unravel the possibility of creating the interface toward c‐ZnO heterogeneous layer, which is a major step for the realization of a better integration of perovskites and charge carrier transport layers.

## Graphical abstract

In situ growth of high‐quality CsPbBr_3_ perovskite single crystal arrays directly on an electron transport layer of cubic zinc oxide (c‐ZnO) via a facile spin‐coating method is presented. CsPbBr_3_ and c‐ZnO can match moderately attributed to the epitaxial lattice coherence. The interface toward c‐ZnO heterogeneous layer is a major step toward the realization of better integration of perovskites and inorganic transport layers.

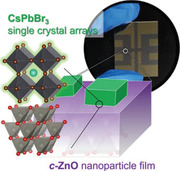

## 1 Introduction

Over the past few years, all‐inorganic lead halide perovskites have attracted tremendous attentions due to their superior advantages including high luminescence efficiency, widely tunable bandgaps, and easy solution processability.^[^ ^1^, ^2^, ^3^ ^]^ Great efforts have been devoted in pursuit of high‐performance optoelectronic devices based on perovskite materials, including solar cells,^[^ ^4^, ^5^, ^6^ ^]^ light‐emitting devices (LEDs),^[^ ^7^, ^8^, ^9^, ^10^, ^11^, ^12^, ^13^ ^]^ photodetectors,^[^ ^14^ ^]^ etc. Regarding to the fabrication of these devices, one of the most widely adopted materials are colloidal perovskite nanocrystals (PeNCs).^[^ ^15^, ^16^, ^17^, ^18^ ^]^ However, despite the efficiency of the PeNC‐based LEDs has been significantly improved since their first appearance, phase impurities and stability issues of the synthesized PeNCs have always been the huge obstacles for large scale industrial applications.^[^ ^17^, ^19^, ^20^, ^21^ ^]^ Specifically, the coexistence of mixed phases of the PeNCs weakens the transport of charge carries and jeopardizes the performance of the devices.^[^ ^19^, ^22^, ^23^, ^24^ ^]^ Moreover, the thermal stability of PeNCs and their resistance to environmental factors like humidity are poor. The functional ligands decorating the outside surface of the PeNCs easily fall off at high temperatures and are highly susceptible to oxygen and light irradiation, leading to the deterioration of PeNCs and device failure.^[^ ^25^, ^26^, ^27^, ^28^, ^29^ ^]^

All‐inorganic perovskite single crystals are considered as promising candidates to tackle all of the above challenges. First, compared with PeNCs, perovskite single crystals have significantly fewer defect‐induced trap states. These trap states are one of the main reasons for the instability of the devices since the trap states will cause nonradiative recombination, resulting in a reduced energy conversion efficiency and a poor performance of the devices.^[^ ^30^ ^]^ Moreover, perovskite single crystals have no inner grain boundaries, which contribute to a higher charge carrier mobility, longer charge carrier lifetime, and thereby greatly improve the device energy conversion efficiency.^[^ ^23^ ^]^ Besides, all‐inorganic perovskites demonstrate a strongly enhanced chemical stability,^[^ ^24^, ^31^ ^]^ which is critical for long‐term operation of devices. Up to now, a wide range of single‐crystalline perovskites has been fabricated via solution processes, including the perovskites that are in nanoscale, bulk counterparts, and other forms.^[^ ^32^ ^]^ Recently, single‐crystalline homo‐epitaxial perovskite thin films made by spin‐coating the precursors of the perovskite onto SrTiO_3_ substrates were demonstrated, which provided a new approach for fabricating single‐crystalline perovskite materials.^[^ ^33^ ^]^ Spin‐coating is a simple, rapid, and inexpensive approach that has been used commercially for device fabrication including depositing polymer films for lithography, unlike these techniques which are usually constrained to ultrahigh vacuum or high temperatures such as molecular beam epitaxy, chemical vapor deposition, and physical deposition.^[^ ^33^ ^]^ However, single‐crystalline perovskite films grown on SrTiO_3_ substrates were not convenient for the fabrication of optoelectronic devices in the typical configuration such as LEDs or solar cells. The typical device configuration of such devices is commonly composed of an emitting layer of perovskite sandwiched between two transport layers connecting to the respective anode and cathode.^[^ ^2^, ^4^, ^5^, ^6^, ^7^, ^8^, ^9^, ^10^, ^11^, ^12^, ^13^ ^]^ Therefore, the synthesized single‐crystalline perovskites grown on top of SrTiO_3_ substrates need to be transferred to be compatible with further device fabrication steps, which would make the entire process complicated and also will cause damages or defects to the perovskite layer. On the contrary, directly growing the perovskite single crystals on transport layers is a promising alternative way to solve this issue, which has always been a paramount technological challenge.

In the present work, we report in situ growth of inorganic perovskite (CsPbBr_3_) single crystal arrays (PeSCAs) on cubic phase ZnO (c‐ZnO) electron transport layers (ETLs) via a facile spin‐coating method without any postannealing steps. The as‐prepared PeSCAs demonstrate phase‐purity and an excellent crystallinity, which are superior to the previous results.^[^ ^9^ ^]^ Through structural characterization, we observe that the (100) plane of cubic CsPbBr_3_ and the (100) plane of c‐ZnO can match moderately in the lattice distance, which facilitates the out‐of‐plane growth of PeSCAs with a preferential crystallographic orientation along the 〈100〉 direction. The epitaxial growth of PeSCAs on c‐ZnO is further confirmed by grazing‐incidence wide‐angle X‐ray scattering (GIWAXS). Compared to the PeNCs counterpart, the PeSCAs show better stabilities of both crystal structure and optical properties in an ambient environment, which is attributed to the absence of grain boundaries and a reduced number of trap states in the PeSCAs. By time‐resolved photoluminescence (TRPL) spectral analysis, PeSCAs reveal less disordered energetic states for exciton diffusion with a faster diffusion rate than that of PeNCs. Moreover, fewer trap states in PeSCAs are confirmed from the longer photoluminescence (PL) lifetime than that of PeNCs as observed in photophysics studies.

## 2 Results and Discussion

### 2.1 Structural Characterizations

**Figure 1:**
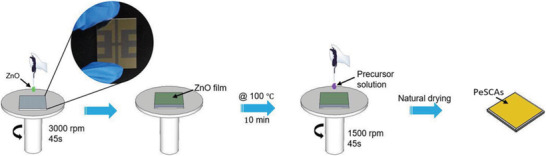
Schematic of the preparation of PeSCAs with the inset showing the substrates used in the experiments.

The PeSCAs were prepared by a two‐step spin‐coating method without a vacuum environment and without annealing process, as schematically illustrated in **Figure** **1**. Specifically, a layer of ZnO was formed by spin‐coating (3000 rpm for 45 s) colloidal ZnO nanoparticles (NPs) on indium tin oxide (ITO) substrates before the growth of the PeSCAs. Then, the substrates were heated at 100 °C for 10 min to remove ethanol and to enhance the homogeneity of the film. The precursor solution of CsPbBr_3_ with an equimolar amount of CsBr:PbBr_2_ was drop‐casted onto the ZnO layer and spin‐coated with 1500 rpm for 45 s. Then, the substrates were positioned in a ventilated cupboard for the drying process, during which the CsPbBr_3_ crystallized on the surface of c‐ZnO NPs. Detailed information on the synthesis process for the precursor solution can be found in the experimental section. At the same time, widely reported CsPbBr_3_ nanocrystals were also prepared to compare with the PeSCAs. Details of the synthesis process of PeNCs are found in the Experimental Section.

**Figure 2:**
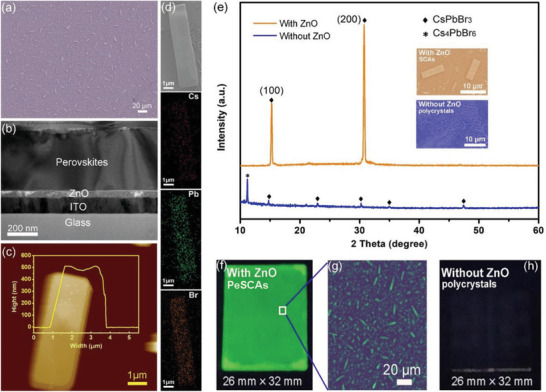
Morphology, structure, and composition analysis of the CsPbBr_3_ SCAs. a) Top and b) sectional SEM images of the CsPbBr_3_ SCAs growing on the ZnO layer drying at room‐temperature in an ambient environment. c) AFM image of the as‐obtained individual single crystal with the overlay image showing the thickness of the sample. d) A typical perovskite single crystal platelet and the corresponding elemental mappings of cesium, lead, and bromine. e) XRD spectra of CsPbBr_3_ with (brown line) and without (blue line) ZnO on the substrates. Upper and lower insets are the SEM images of perovskites on substrates with and without ZnO. f,h) Real optical images of SCAs growth on the substrate with and without ZnO under ultraviolet (UV) light (365 nm) with g) the zoom‐in view of the white box zone in (f).

The morphology of as‐obtained PeSCAs can be observed by scanning electron microscopy (SEM) (**Figure** **2**a), showing that the substrates are covered with PeSCAs, which are rectangular microplatelets of 6–10 µm in length and 2–3 µm in width. The SEM cross‐sectional view (Figure 2b) shows that the average thickness of these microplatelets is ≈400 nm and that there are no observations of prominent interface gaps at the interface of the single crystals and the ZnO. To probe the element distribution of the cross‐section, the corresponding energy dispersive X‐ray (EDX) elemental mapping and spectra of the perovskites/ZnO heterojunctions are shown in Figures S1 and S2 in the Supporting Information where the element ratios of Cs, Pb, and Br, are close to the stoichiometry of CsPbBr_3_. Moreover, the thickness of the PeSCAs was further obtained by an arbitrary selection of four different microplatelets and characterized by atomic force microscopy (AFM) (Figure 2c; Figure S3, Supporting Information). The AFM results show that the thickness of as‐prepared PeSCAs is ranged from 400 to 500 nm. The composition and crystal structure analysis (Figure 2d,e) of the CsPbBr_3_ SCAs were performed with EDX and X‐ray diffraction (XRD). For XRD measurements, we spin‐coated the perovskite precursors on the ZnO‐covered substrates and ZnO‐free substrates as reference samples, respectively. The obtained cubic crystalline structure for CsPbBr_3_ SCAs on the ZnO‐covered substrates is indicated by the upper XRD pattern in Figure 2e (brown line), where two distinct characteristic peaks at 15.3° and 30.8° are assigned to the typical (100) and (200) crystal planes of CsPbBr_3_, respectively. In comparison, XRD patterns of the reference sample show a clear observation of diffraction from an impurity (Cs_4_PbBr_6_)^[^ ^34^, ^35^ ^]^ and multiple plane orientations, suggesting a polycrystalline nature of the samples. In more detail, the samples on the ZnO‐free substrates are Cs_4_PbBr_6_‐dominated polycrystals. In addition, the intensities of these peaks are much lower. The XRD results suggest a good crystallinity of the grown CsPbBr_3_ SCAs with a pure phase on the ZnO layer, corresponding to the EDX spectrum (Figure S2, Supporting Information) and elemental mappings (Figure S1, Supporting Information; Figure 2d). The results of EDX and XRD are also confirmed by the optical images of the PeSCAs and the reference sample under UV light. There is a green light emission from the PeSCAs (Figure 2f,g) and almost no radiation from the reference sample, which is consistent with previous reports that CsPbBr_3_ is a green‐emission semiconductor and Cs_4_PbBr_6_ is a nonluminous insulator.^[^ ^7^, ^8^, ^9^, ^10^, ^11^, ^12^, ^13^, ^36^, ^37^ ^]^

**Figure 3:**
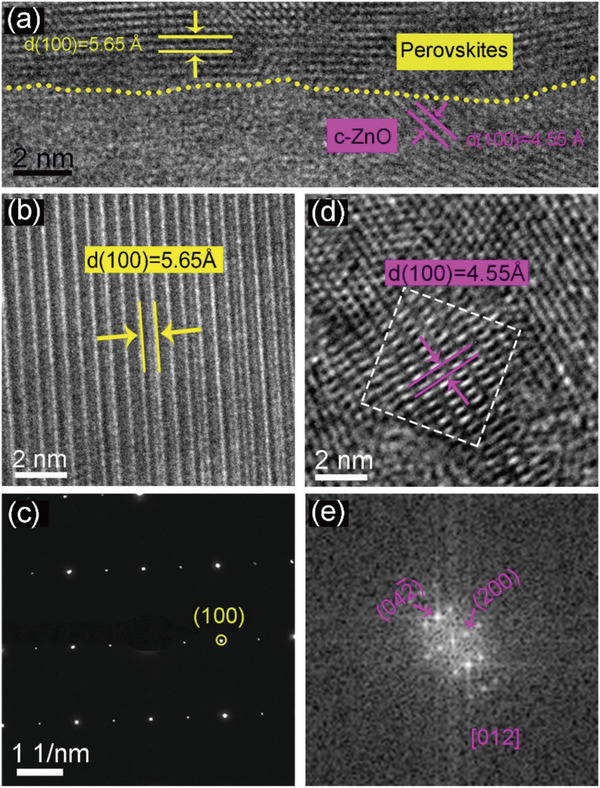
a) HRTEM image of the interface region of perovskites/c‐ZnO. b) HRTEM image of perovskites and c) the corresponding SAED pattern. d) HRTEM image of c‐ZnO and e) the corresponding FFT.

**Figure 4:**
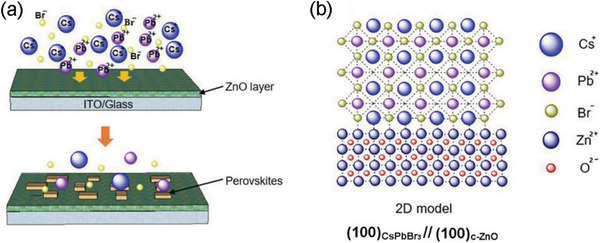
a) Schematic diagram of the epitaxial growth of CsPbBr_3_ SCAs on c‐ZnO surface. b) 2D model atomic crystal heterojunction structure of the (100) plane of CsPbBr_3_ and the (100) plane of c‐ZnO.

The crystal quality of the PeSCAs and their heterointerface with the ZnO layer were also investigated by high‐resolution transmission electron microscopy (HRTEM) and selected area electron diffraction (SAED). The cross‐sectional observation was enabled by a focused ion beam (FIB) cutting. It is noted that c‐ZnO NPs are formed stochastically in the spin‐coated ZnO layer and the high quality of the perovskites/c‐ZnO interfaces are demonstrated in the HRTEM image (which was obtained 30 days after perovskites/c‐ZnO heterojunctions were prepared) (**Figure** **3**a), where an abrupt interface at an atomic level is witnessed by the yellow dotted line, indicating epitaxial orientation alignment at two dominant facets.^[^ ^38^ ^]^ Moreover, the distinct lattice fringe of ZnO NPs (including c‐ZnO) in the image indicates excellent structural stability of the ZnO NPs beneath CsPbBr_3_ perovskites, which is corresponding to the XRD spectra of ZnO NPs in Figure S5 in the Supporting Information. Figure 3b shows the HRTEM image of the pure perovskite region, where the clear lattice fringes suggest high crystal quality of PeSCAs. The plane with the lattice distance of 5.65 Å is indexed to the (100) plane for the perovskites. The corresponding SAED pattern (Figure 3c) confirms a single‐crystalline growth of the perovskites in the cubic crystal structure. The HRTEM image of a pure c‐ZnO region indicated by the white dotted box is illustrated in Figure 3d. The lattice fringes of 4.55 Å spacing are ascribed to (100) planes, which is in accordance with the cubic crystalline ZnO consistent with the corresponding Fast Fourier transformations (FFTs) (Figure 3e). The in situ heteroepitaxial growth mechanism of CsPbBr_3_ SCAs and the corresponding atomic schematic are illustrated in **Figure** **4**a. A thin layer of perovskite crystallites nucleates on the surface of the ZnO NPs in the initial stage of the growth. Due to the cubic blende structure of ZnO (as shown in Figure 3d,e), Cs^−^, Pb^2+^, and Br^−^ being in the precursors bond to the outside surface of the ZnO to form a new perovskite layer,^[^ ^39^, ^40^ ^]^ following the cubic structure and crystallographic orientation of the ZnO structure (Figure 4b).^[^ ^41^ ^]^ The newly formed perovskite crystals stack layer‐by‐layer and continue to grow (Figure 4b). The low lattice mismatch between CsPbBr_3_ and c‐ZnO facilitates the epitaxial growth of CsPbBr_3_ on c‐ZnO. The lattice constants measured in our experiment are 5.65 and 4.55 Å (Figure 3a–d) for (100) _CsPbBr3_ and (100) _ZnO_, respectively, which are close to the theoretical values of 5.605 and 4.6 Å.^[^ ^42^ ^]^ This results in a crystal facet mismatch of 19% allowing for epitaxy growth, which is similar to the reported single‐crystalline heterojunction of ZnO/ZnS.^[^ ^43^ ^]^ The matched epitaxial layers make it possible to nucleate on the crystalline ZnO NPs. Crystallization will end after the subsequent evaporation of the solvent. We also observed the crystallization process of the PeSCAs under UV light (365 nm), as shown in Figure S6 in the Supporting Information. The whole crystallization process lasted for ≈20 min. The crystallization started at the edge and ended in the middle. According to the crystallographic principles,^[^ ^33^ ^]^ the very thin supersaturated solution layer on the edge of the ZnO covered substrates initialized the nucleation from the spin‐coated solution. The lower thickness of the solution at the edge is caused by surface tension and causes a faster solvent evaporation rate at the edge as compared with the film center. The concentration gradient from the edge to the middle promotes nucleation of the material onto the c‐ZnO covered substrates.

As a comparison, hexagonal crystal phases of ZnO (h‐ZnO) (Figure S7, Supporting Information) also exist in the ZnO NPs besides c‐ZnO. It is difficult for h‐ZnO to induce the formation of cubic CsPbBr_3_ due to the large lattice mismatch. It is known that the lattice parameters measured for CsPbBr_3_ and wurtzite phase ZnO^[^ ^42^ ^]^ are 5.65 and 3.25 Å, respectively. The lattice mismatch between (100) CsPbBr_3_ and hexagonal (10$\bar{1}$0) ZnO is as high as 42%. As a result, the growth of PeSCAs cannot be initiated by h‐ZnO NPs. In order to verify this speculation, we spin‐coat the perovskite precursors on standard hexagonal  〈0001〉 single‐crystalline ZnO substrates. XRD results (Figure S8, Supporting Information) show a prominent ZnO pattern^[^ ^44^ ^]^ with mixed peaks resulting from CsPbBr_3_ and Cs_4_PbBr_6_,^[^ ^37^ ^]^ which suggests a multiphase and polycrystalline character of the perovskites on these substrates. It is noted that there is almost no green light emission from the perovskites coated on hexagonal ZnO substrates, indicating the domination of Cs_4_PbBr_6_^[^ ^35^, ^37^ ^]^ of the sample on substrate although with a small mixed proportion of CsPbBr_3_.

**Figure 5:**
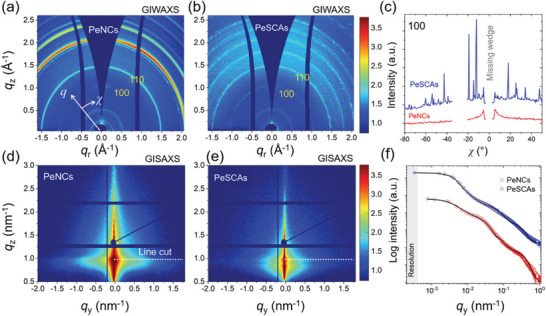
2D GIWAXS data of a) PeNCs and b) PeSCAs with indexed (100) and (110) Bragg peak positions. c) Corresponding azimuthal integrals of the (100) Bragg peak as indicated in (a) and (b). 2D GISAXS data of d) PeNCs and e) PeSCAs. f) Corresponding horizontal line cuts at the Yoneda peak positions as indicated in (d) and (e).

To further verify the crystalline nature of CsPbBr_3_ SCAs, we also characterize the samples with GIWAXS and grazing incidence small‐angle X‐ray scattering (GISAXS), including measurements on a reference sample of spin‐coated PeNCs. **Figure** **5**a,b shows the corrected 2D GIWAXS data for PeNCs and PeSCAs in which the bright rings in the GIWAXS patterns indicate the presence of crystallites with a random orientation for both PeNCs and PeSCAs. The tube cuts performed around the (100) Bragg peak (0.9 Å^−1^ < *q* < 1.1 Å^−1^) suggest that only PeNCs are well aligned statistically along the substrate, with an enhanced Bragg peak intensity distributed around the *χ* = 0° position as seen in Figure 5c. In contrast, bright intensity spots are seen along with the Bragg peak rings in the GIWAXS pattern of the PeSCAs due to the presence of larger individually oriented crystals. The distribution of the scattering spots along the (100) Bragg peak of PeSCAs is also illustrated in Figure 5c, which suggests that the single crystals demonstrate a strong specific scattering and that they are not perfectly aligned with respect to the substrate. These findings are in good agreement with previous studies.^[^ ^45^, ^46^ ^]^ Moreover, from an azimuthal integration of *q* in a range of 0.4 Å^−1^ < *q* < 2.7 Å^−1^, as seen in Figure S9a in the Supporting Information, the Bragg peaks of PeNCs and PeSCAs are all matching with the theoretical *q* values, which are corresponding to the specific lattice distances. However, PeSCAs demonstrate split peaks, which is due to having slightly different crystal lattices or a reduction of symmetry of the phases. Considering the large size of single crystals compared with PeNCs, the number of single crystals illuminated by X‐ray is quite limited contributing to the scattering intensity. The GIWAXS pattern for the bare ZnO layer on the ITO substrate is also provided as seen in Figure S9b in the Supporting Information to identify the contribution from the substrate. In addition, 2D GISAXS data are shown in Figure 5d,e for both, PeNCs and PeSCAs, in which the corresponding horizontal line cuts are indicated by the white dash‐line position. The line cut data are shown in Figure 5f. The vertical line cuts around *q*_y_ = 0 nm^−1^ are also provided in Figure S10 in the Supporting Information. The horizontal line cut positions (critical angle position) of PeNCs, as well as PeSCAs, are confirmed according to the scattering length density calculation at around 0.21° with the X‐ray photon energy = 11.7 keV. The PeSCA samples reveal a more apparent Yoneda peak signal at the critical angle position than the PeNCs sample due to a higher surface roughness caused by the single crystals.^[^ ^47^ ^]^ The crystal size distribution for PeNCs and PeSCAs are analyzed in the framework of the distorted wave Born approximation using the local monodisperse approximation by using a model with 3 cylinders of different diameters at different distances (as seen in Table S2 in the Supporting Information).^[^ ^48^ ^]^ The larger crystals have an average size of around 0.7 µm. Notably, larger‐sized single crystals (over 1 µm) as seen in the SEM images, cannot be detected in the GISAXS measurements due to the resolution limitation, which is related to the X‐ray photon energy and the sample‐detector distance. However, their presence is seen in the intensity increase in the resolution limit.

### 2.2 Structural and Optical Stabilities

**Figure 6:**
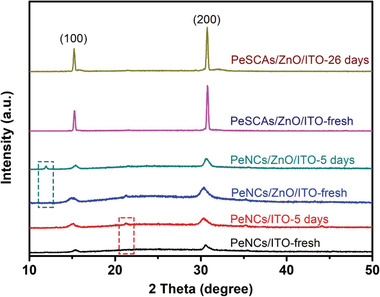
Structure stability investigation. XRD patterns of PeSCAs on substrates versus PeNCs with various aging durations in an ambient environment.

Resistance to room temperature and humidity of the as‐prepared PeSCAs is investigated. For comparison, reference samples of PeNCs were also prepared on the same type of ITO glass. Both samples were stored in the same ambient environment (23.4 °C and 70% relative humidity (RH)) without inert gas protection. As shown in **Figure** **6**, the XRD intensities and peak positions of the PeSCAs remain almost unchanged after 26 day storage without phase transitions and impurities formation, whereas the PeNCs exhibit an apparent phase collapse and a variation after only 5 days of storage. This result proved that PeSCAs have a long‐term structure stability. Moreover, as a contrast, we also spin‐coated a layer of bare ZnO NPs beneath the PeNCs, indicated by blue and blue‐green lines in Figure 6. As marked in the rectangular dashed boxes, both PeNCs on ZnO/ITO and PeNCs on ITO exhibit a fast degradation during 5 day storage, and it is noted that the PeNCs on ITO have more XRD peaks revealing an inferior crystal phase of PeNCs on ITO. The results reveal that PeSCAs have superior structural purity and stability than the PeNCs.

**Figure 7:**
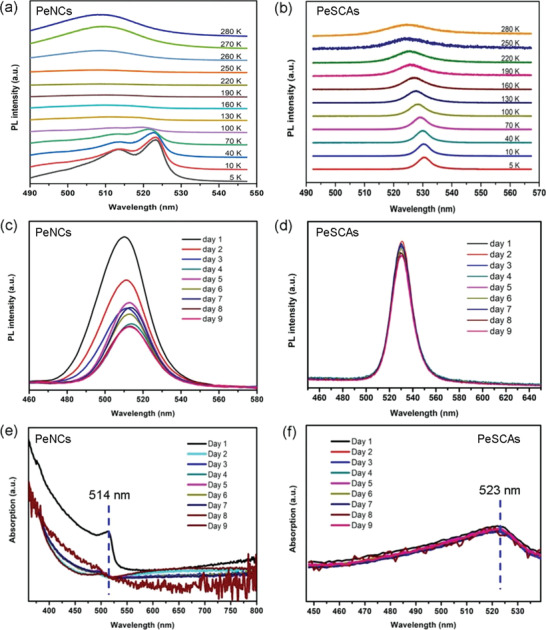
Stability investigation of structure and optical characterization of CsPbBr_3_. Temperature‐dependent photoluminescence (TDPL) of a) PeNCs and b) PeSCAs. Room temperature photoluminescence (RTPL) of c) PeNCs and d) SCAs for 9 days. Absorption of e) PeNCs and f) PeSCAs within 9 days, respectively.

To unravel the hidden mechanism of radiative PL efficiency of PeSCAs, temperature‐dependent PL measurements are carried out in this study as shown in **Figure** **7**. The PeNCs were spin‐coated on ITO substrates at 3000 rpm for 45 s. Figure 7a,b shows temperature‐dependent PL data of the PeNCs and PeSCAs, both recorded from 10 to 280 K. In Figure 7a, the spectrum from PeNCs at 5 K is dominated by two peaks, centered at 513 and 523 nm, respectively. With the increase of temperature, both peaks blue‐shift and their intensity gradually decreases and fades away at 160 and 100 K. In addition, a new emission peak appears form 190 K on at the shorter wavelength and dominates the spectrum until room temperature. It was reported that the peaks recorded at 523 nm (at 5 K) and 513 nm (at 5 K) are derived from bound exciton luminescence and free exciton luminescence, respectively.^[^ ^49^ ^]^ At low temperature, the number of bound excitons is much larger than that of free excitons. Therefore, bound excitons contribute to a higher intensity of the peaks related to the luminescence at 523 nm (5 K). The decrease of PL emissions (for both peaks at 523 and 513 nm) at higher temperatures (from 100 to 190 K) is related to exciton dissociation.^[^ ^50^ ^]^ The emerging PL peak with a shorter wavelength is attributed to band‐to‐band recombination because the luminescence from band‐to‐band recombination could also be observed at high temperature.^[^ ^49^ ^]^ With the rise of temperature, the luminescence intensity is not affected by exciton decomposition. Moreover, the emerging PL peak and the blue‐shift in the temperature range from 190 to 280 K are possibly due to a structural phase transition.^[^ ^51^ ^]^ For PeSCAs (Figure 7b), the PL peak originates from the free excitons and the observation of decrease of the emission peaks with the increase of temperature is in agreement with the collapse of free excitons.^[^ ^50^ ^]^ For both PeNCs and PeSCAs, the gaps between the valence band and the conduction band increase with the rise of temperature, resulting in a larger bandgap in the electron transition process. Thus, the blue‐shift is observed from 5 to 280 K.^[^ ^52^, ^53^, ^54^ ^]^

The PL stabilities of PeSCAs and PeNCs at room temperature are evaluated at 365 nm excitation in the ambient environment (22.0 °C and 75% RH), as shown in Figure 7c,d. The PL intensities of PeNCs decreases dramatically in the first 4 days and continues to decay after a slight rise, which is similar to previous observations.^[^ ^29^ ^]^ The phenomenon was attributed to more and more ligands lifting off from the surface of PeNCs as time prolonged, which resulted in increased trap states and reduced PL intensity drop due to the capture of excitons by the ligands traps.^[^ ^29^ ^]^ Besides, the PeNCs aggregated into larger nanocrystals^[^ ^28^, ^29^ ^]^ as shown in Figure S11 in the Supporting Information. Compared to the fresh‐prepared PeNCs, the crystal sizes rise to 10 times larger values. The aggregation process causes the decreased density of trap states and due to the reduction of the surface area to volume ratio, a gentle rise of the PL intensity is observed. Correspondingly, the emission peak at 510 nm shifts to 513 nm on the fifth day. However, once the PeNCs aggregated into larger ones, their quantum confinement effect gradually disappears and the PL intensity of PeNCs decreases again. This result is consistent with the PL quantum yield (PLQY) test (black line in Figure S12 in the Supporting Information). On the contrary, the PeSCAs kept a favorable PL stability against the moisture at room temperature with a negligible PLQY variation of 0.8–1% (red line in Figure S12 in the Supporting Information). This behavior is explained by having fewer recombination channels and less trap density in PeSCAs than PeNCs. In comparison, the PeNCs possess a larger number of grain boundaries thereby causing more nonradiative recombination.^[^ ^30^ ^]^ In addition, an increased density of trap states causes an inferior optical performance owing to the peeling off of the ligands during the aging process.^[^ ^21^ ^]^ However, it is an opposite case for PeSCAs since the capping ligands are not employed for the growth of the single crystals, and thus they would not compromise the stability of PeSCAs. Room temperature absorption spectra for PeNCs and PeSCAs are shown in Figure 7e,f. The PeNCs and PeSCAs show absorption at respective first exciton peaks in the range of ≈514 and ≈523 nm. The PeNCs exhibit a drop of absorption intensity at the peak position with a 9 day measurement, whereas PeSCAs remain almost unchanged in intensity under the same test condition. Consequently, PeSCAs have better stability of their absorption spectral properties over PeNCs.

### 2.3 Exciton Dynamics Analysis

**Figure 8:**
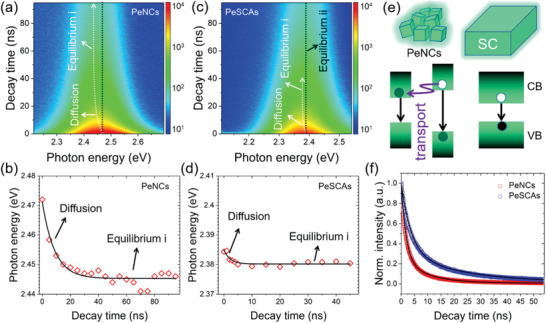
2D pseudocolor plot of room‐temperature transient PL (TRPL) spectra of a) CsPbBr3 PeNCs and c) single‐crystalline arrays (PeSCAs). The color scale stands for the number of photons. The excitation wavelength is 405 nm. Corresponding fitted photon energy as a function of decay time for b) PeNCs and d) PeSCAs, respectively. e) Schematic of the energy transport in PeNCs resulting in energy peak shift, while, this rarely happens among single crystal array due to their large surface to surface distance. f) Time‐resolved PL data of PeNCs (red circle) and PeSCAs (blue circle) fitted by a double exponential decay function (solid lines).

To evaluate the energetic disorder in PeNCs and PeSCAs, the dynamics of excitons for radiative recombination in the two samples are studied by TRPL with corresponding spectral mapping. **Figure** **8**a,c shows the temporal PL spectra with the white arrows indicating the radiative recombination centers. The radiative recombination centers initially red‐shift due to the exciton diffusion from higher energy levels to lower energy levels. Tracking these peak shifts provides a feasible way to describe the inner energetic disorder for the exciton recombination. The exciton diffusion rate is fitted by Equation (1)^[^ ^55^ ^]^

(1) $$\begin{array}{c} E\;\left(t\right)=E_{0}\;+\Delta E\exp\left(-k_{\Delta E}t\right) \end{array}$$

in which *E*(*t*) is the real‐time energy peak position, and *E*_0_ is the final equilibrium energy state (equilibrium state *i* in Figure 8 a,c) for exciton recombination. Δ*E* is the energy gap between the initial excited state and equilibrium state *i*, which can be used to describe the energetic disorder of the film. It is also considered to be the driving force for the exciton diffusion. The exciton diffusion rate is denoted by *k*_Δ_ *_E_*, and the fitted values are (0.113 ± 0.005) and (0.455 ± 0.055) ns^−1^ for PeNCs and PeSCAs (Figure 8b,d), respectively. The results show that the PeSCAs possess a much narrower energy state distribution than that of PeNCs,^[^ ^56^ ^]^ whereas the exciton diffusion rate of PeSCAs is significantly higher than that of PeNCs. As shown in the schematic diagram in Figure 8e, the bandgaps in the PeSCAs are all close to the theoretical value of the bulk materials’ bandgap with extremely reduced quantum confinement effects (size effect). Thus, the bandgaps are narrower distributed in PeSCAs than in PeNCs, which matches well with the spectra in Figure 5. Moreover, the exciton recombination in PeSCAs mainly occurs in each single crystals with little energy transfer from one to another via resonance or tunneling due to the long adjacent distance among neighboring single crystals. The PeSCAs also show a blue‐shift feature as observed by tracking the emission peaks (Figure S13, Supporting Information), corresponding to the long‐lifetime excitons in PeSCAs. The energy transfer is completely blocked and thus the radiative recombination center maintains at the equilibrium state ii for a longer time as indicated by the dark dash‐line in Figure 8c.

Besides the time‐resolved spectral analysis, we also track the exciton lifetime by tracking the equilibrium state excitons of PeSCAs and PeNCs, respectively as shown in Figure 8f. A double exponential decay function is used to fit the decay curves. The results are provided in **Table** **1** and Table S3 in the Supporting Information. In which the *τ*_1_ and *τ*_2_ are considered to relate to the hole‐trap states and electron‐trap state, respectively. Longer lifetimes mean less corresponding trap densities. PeSCAs reveal fewer trap densities than PeNCs, with significant implications for those higher efficient optoelectronic applications.

**Table 1:** Fitting data from the decay curves of PeNCs and PeSCAs

Sample	*τ*_1_ [ns]	*τ*_2_ [ns]
PeNCs	1.808	9.561
SCAs	3.637	20.9

## 3 Conclusion

In summary, herein direct growth of PeSCAs on an electron transport layer of c‐ZnO is achieved via a facile spin‐coating method. The GIWAXS measurements confirm the single‐crystalline properties of the as‐obtained perovskite arrays, attributed to the epitaxial lattice coherence of (100)_CsPbBr3_∥(100)_c‐ZnO_. A growth mechanism is proposed for the formation of stable cubic phased PeSCAs by analyzing the mismatch degree between epitaxial single‐crystalline arrays and the underneath c‐ZnO. Furthermore, the PeSCAs show an excellent structure and optical stability, which is superior to state‐of‐the‐art PeNCs, due to the presence of high‐quality single‐crystalline properties. The achievement of an atomically smooth and abrupt interface at the c‐ZnO heterogeneous layer is a major step forward, as this paves the way to an efficient combination of perovskites and n‐type semiconducting ETL, which is promising to realize various applications.

## 4 Experimental Section

##### Precursor Solution Preparation

Equimolar CsBr (99.5%, Aladdin) and PbBr_2_ (99.0%, Aladdin) were dissolved in dimethyl sulfoxide (DMSO, >99% Shanghai Ling Feng Chemical Reagent Co., Ltd.) at 50 °C to obtain 0.4 m solution. After stirring, MeCN (>99% Shanghai Ling Feng Chemical Reagent Co., Ltd.) was dropped into the previous solution until the solution turned pale yellow. Then, the as‐obtained solution was heated at 50 °C for 24 h.

##### PeNCs Preparation

The CsPbBr_3_ NCs were prepared by using the hot injection technique previously described in the literature.^[^ ^57^ ^]^ Briefly, for the Cs‐oleate preparation, Cs_2_CO_3_ (0.0814 g, Aldrich, 99.9%) was loaded into 10 mL 3‐neck flask with octadecene (10 mL, Sigma‐Aldrich, 90%) and oleic acid (1 mL, OA, Sigma‐Aldrich, 90%), stirred for 10 min at room temperature, and then heated under N_2_ to 150 °C before injection. Simultaneously, 0.414 g PbBr_2_ was loaded into 25 mL 3‐neck flask and then octadecene (30 mL, Sigma‐Aldrich, 90%), oleylamine (3 mL, OLA, Sigma‐Aldrich, 90%), and OA (3 mL, OLA, Sigma‐Aldrich, 90%) were injected. After stirring, the system was heated to 180 °C under N_2_. Cs‐oleate solution (3 mL, prepared as described above) was quickly injected and, 5 s later, the reaction mixture was cooled by the ice‐water bath. After filtering and purification, the 5 mg mL^−1^ methylbenzene‐based CsPbBr_3_ nanocrystal solution was prepared for use.

##### ZnO NPs

The ZnO NPs (20 mg mL^−1^) were purchased from Suzhou Xingshuo Nanotech Co., Ltd. for direct use without further purification

##### Substrates Preparation

ITO substrates (purchased from Ying Kou You Xuan Trade Co., Ltd.) were cleaned by the following procedures in sequence for use: ITO cleaning fluid, deionized water, acetone, isopropanol, with 70% power ultrasound for 10 to 20 min at each step, as the previous methods.^[^ ^11^, ^13^ ^]^

##### Hexagonal ZnO Single Crystal Substrates Preparation

The substrates were purchased from Hefei Crystal Technical Material Co., Ltd. The 0.5 cm × 0.5 cm × 0.5 mm 〈0001〉 single‐crystalline hexagonal ZnO substrates were cleaned with acetone, ethanol, and water in an ultrasonic bath for 15 min, respectively, and then treated in a UV‐Ozone cleaner for 20 min prior to use.

##### Materials Characterizations

XRD measurements were carried out by an X‐ray diffractometer (Rigaku Smartlab). The surface‐section/cross‐section morphologies of the perovskite arrays were characterized using field‐emission scanning electron microscopy (FESEM, Gemini SEM 300). Transition electron microscope (TEM, FEI Talos F200X) and the HRTEM (FEI, Tecnai F30) were employed to analyze the structures and morphologies of the perovskite NCs and ZnO NPs. Grazing‐incidence X‐ray scattering experiments were carried at the DESY synchrotron (beamline P03)^[^ ^58^ ^]^ with an X‐ray photon energy of 11.7 keV and an incident angle of 0.4°. The sample detector distances were 2324 mm for GISAXS and 164 mm for GIWAXS. For GISAXS measurements, a 2D detector Pilatus 1M (Dectris Ltd.) and, for GIWAXS measurements, a 2D detector Pilatus 300k (Dectris Ltd.) were used. For GIWAXS data, intensity corrections and reshaping steps to account for the inaccessible *q*‐space were performed using the MATLAB‐based software GIXSGUI.^[^ ^59^ ^]^ The PL spectra (perovskite NCs) and QY measurement were conducted with an absolute PL quantum yield spectrometer (Hamamatsu, C11347). The PL spectra of perovskite arrays were performed by Ocean Optics QE65pro equipped with 360 nm laser. Absorption spectra were recorded with an UV/VIS/NIR spectrometer (lambda 950). The TRPL measurement was carried out by Fluo Time 300 fluorescence lifetime spectrometer (PicoQuant) with an excitation wavelength of 405 nm. TDPL was characterized by Montana fusion f2, with a laser (Ar+, 488 nm).

## Conflict of Interest

The authors declare no conflict of interest.

## Supporting information

Supporting Information
