# Supplementary material for: In Situ Growth of All‐Inorganic Perovskite Single Crystal Arrays on Electron Transport Layer
Source: Adv Sci (Weinh). 2020 Apr 22;7(11):1902767. doi: 10.1002/advs.201902767 (PMC7284191; doi:10.1002/advs.201902767)
Supplement: Supplementary file 1 — Supporting Information [file ADVS-7-1902767-s001.pdf]

Copyright WILEY-VCH Verlag GmbH & Co. KGaA, 69469 Weinheim, Germany, 2020.

**In-situ Growth of All-inorganic Perovskite Single Crystal Arrays on Electron Transport Layer**

*Xiaobing Tang, Wei Chen, Dan Wu*, Aijing Gao, Gaomin Li, Jiayun Sun, Kangyuan Yi, Zhaojin Wang, Guotao Pang, Hongcheng Yang, Renjun Guo, Haochen Liu, Huaying Zhong, Mingyuan Huang, Rui Chen, Peter Müller-Buschbaum, Xiao Wei Sun, and Kai Wang**

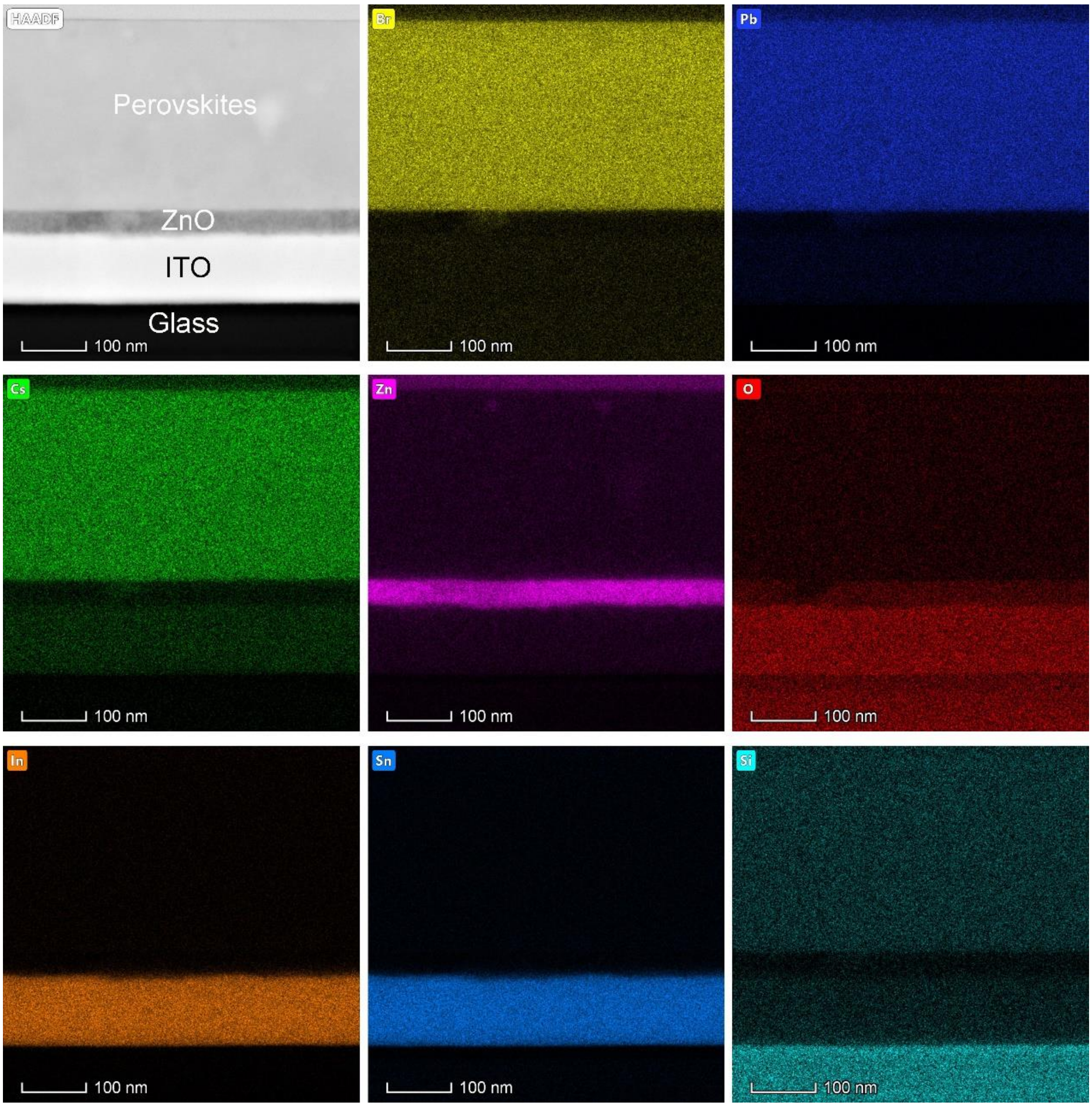

**Figure S1.** Scanning transmission electron microscopy (STEM) images and corresponding elemental mappings of $CsPbBr_3$/ZnO heterojunctions on ITO glass.

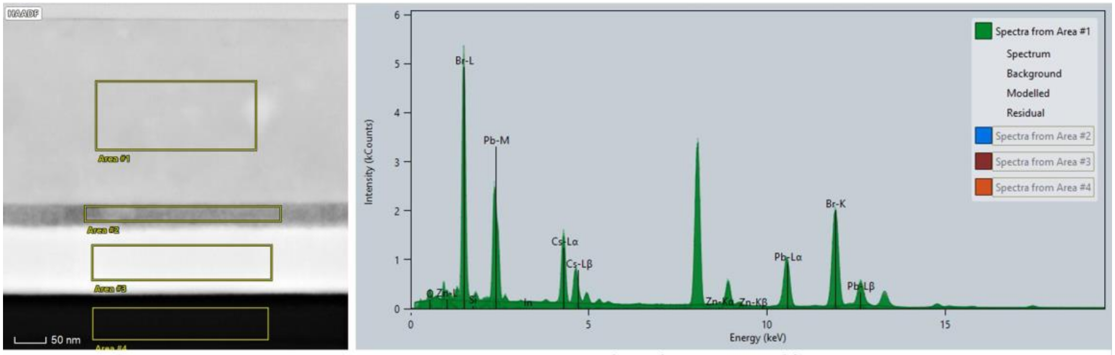


| Z | Element | Family | Atomic Fraction (%) | Atomic Error (%) | Mass Fraction (%) | Mass Error (%) | Fit error (%) |
|---|---|---|---|---|---|---|---|
| 8 | O | K | 4.77 | 0.64 | 0.69 | 0.05 | 2.61 |
| 14 | Si | K | 0.85 | 0.21 | 0.22 | 0.05 | 6.84 |
| 30 | Zn | K | 0.49 | 0.09 | 0.29 | 0.04 | 2.52 |
| 35 | Br | L | 56.28 | 10.60 | 40.63 | 6.23 | 0.08 |
| 49 | In | L | 0.00 | 0.00 | 0.00 | 0.00 | 0.00 |
| 50 | Sn | L | 0.00 | 0.00 | 0.00 | 0.00 | 0.00 |
| 55 | Cs | L | 18.22 | 3.10 | 21.88 | 2.86 | 0.12 |
| 82 | Pb | L | 19.39 | 3.36 | 36.30 | 4.89 | 0.04 |

**Figure S2.** Energy dispersive X-ray (EDX) spectrum of $CsPbBr_3$/ZnO heterojunctions on ITO glass (left) and related areas of measurements (right).

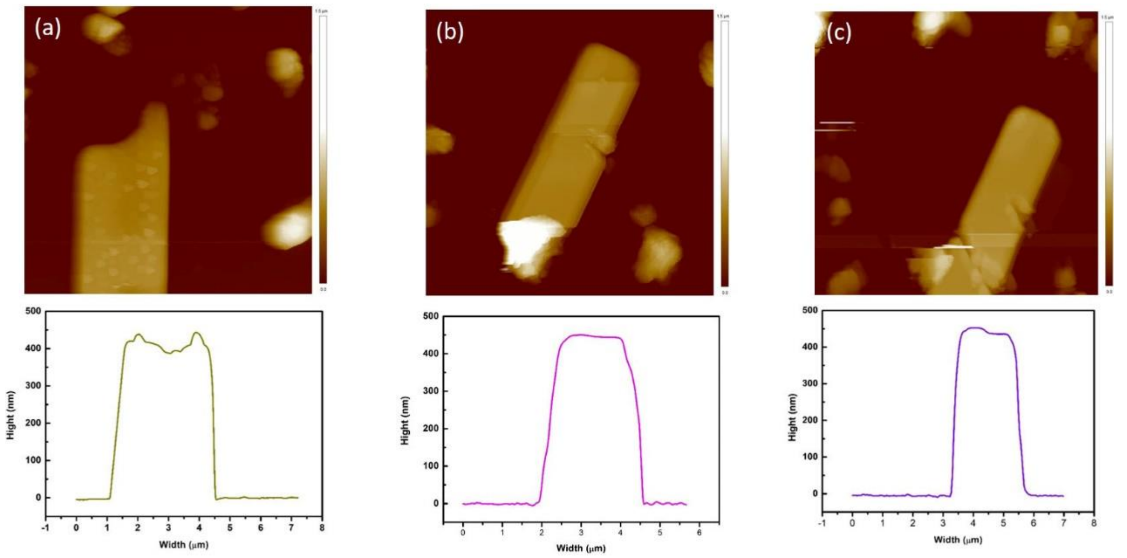


**Figure S3.** Atomic force microscopy (AFM) images of arbitrary three single crystals in as-prepared $CsPbBr_3$ single crystal arrays (SCAs).

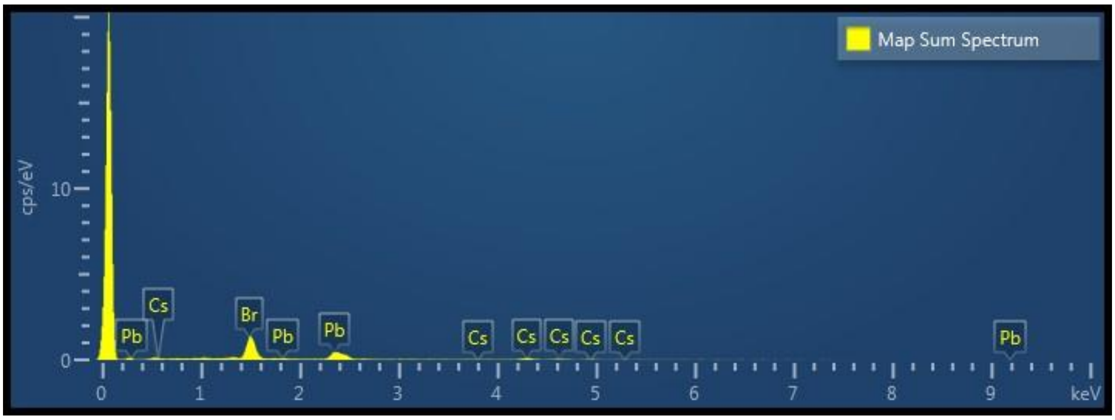


**Figure S4.** Energy dispersive X-ray (EDX) spectrum of as-prepared $CsPbBr_3$ single crystal arrays (SCAs).

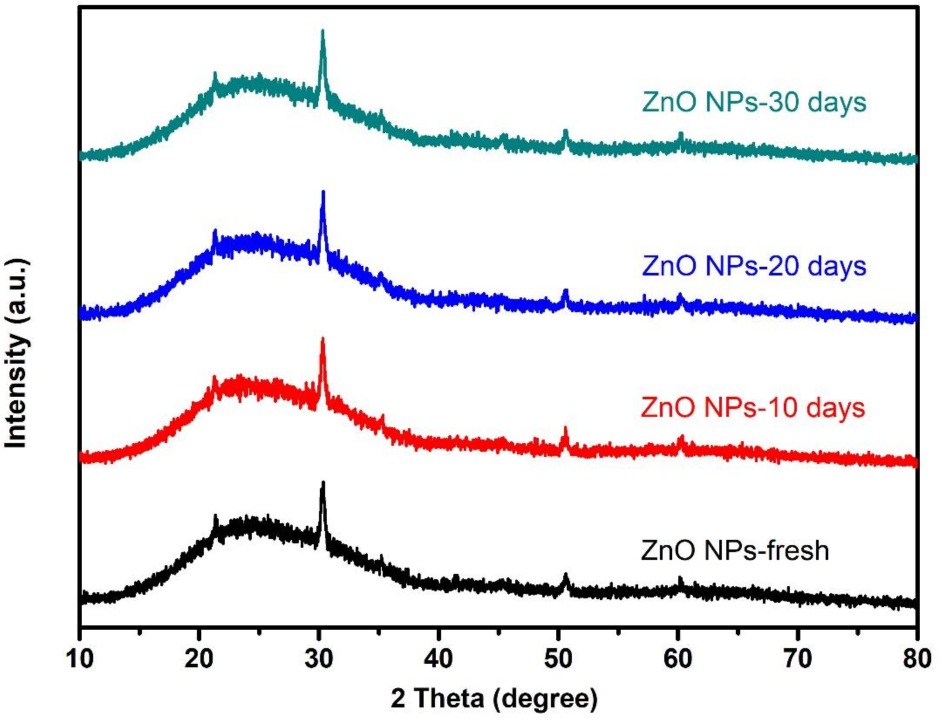


**Figure S5.** XRD patterns of fresh ZnO NPs films (black) on ITO substrates and films which had been stored in the ambient environment for 10 (red), 20 (blue) and 30 (peacock blue) days.

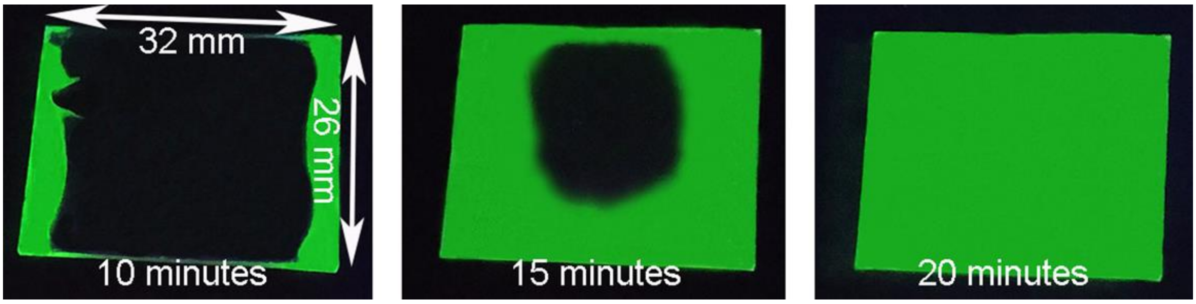


**Figure S6.** Optical images of the induced-growth $CsPbBr_3$ films under purple light (365 nm) with various stages (1500 rmp/45s).

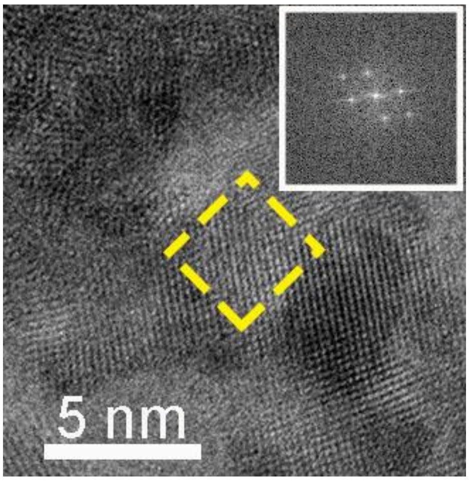


**Figure S7.** High-resolution transmission electron microscopy (HRTEM) image of hexagonal-ZnO (h-ZnO) NPs and Fast Fourier transformations (FFTs) (inset) of the regions indicated in the HRTEM images.

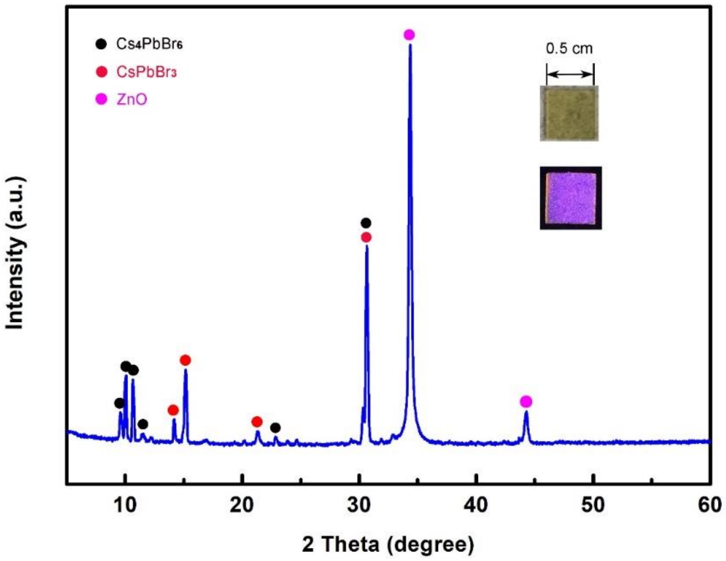


**Figure S8.** XRD data of perovskite precursor on hexagonal ZnO substrates with index peaks. Inset: optical images of precursor on hexagonal ZnO under white light (upper) and purple light (lower) after drying. The size of the substrates is 1 cm ×1 cm × 0.5 mm.

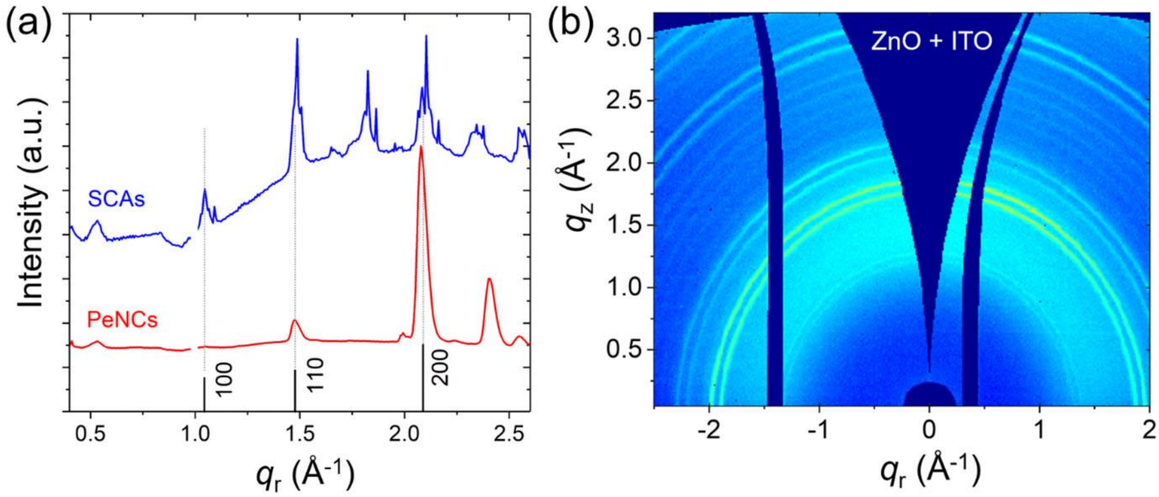


**Figure S9.** a) Cake cut from 2D GIWAXS data of PeNCs (red) and PeSCAs (blue). b) 2D GIWAXS data of the substrate (ZnO on ITO glass).

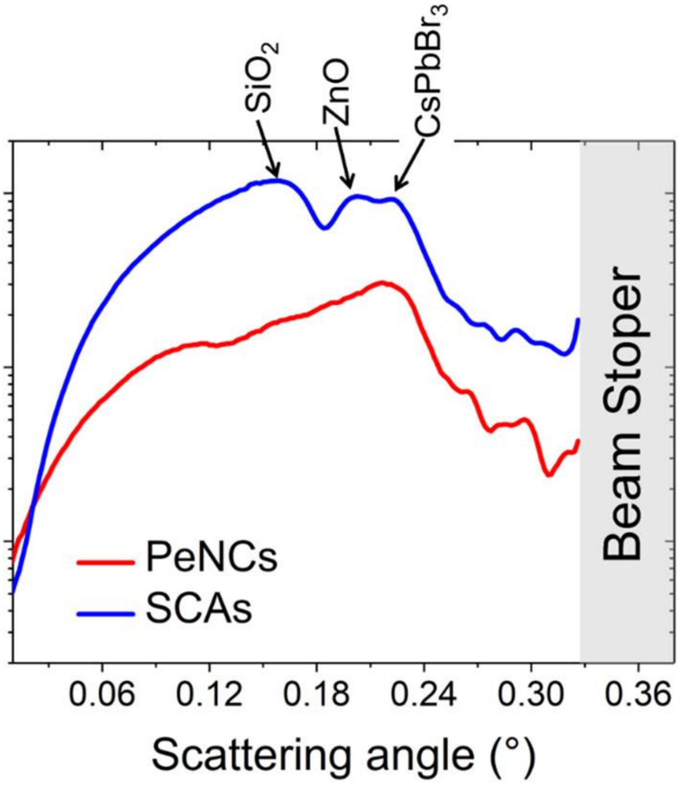

**Figure S10.** Vertical line cuts at $q_y$=0 of 2D GISAXS data for PeNCs (red) and SCAs (blue). The position of the Yoneda peaks for $SiO_2$, ZnO and perovskite are shown by arrows.

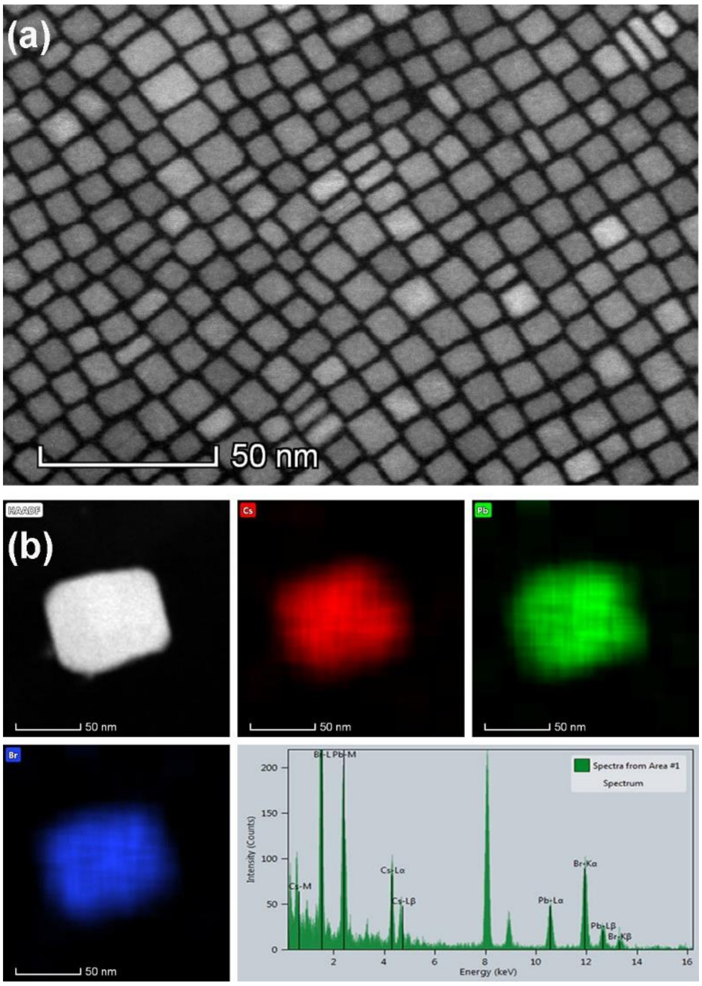


**Figure S11.** a) TEM image of fresh prepared $CsPbBr_3$ NCs with an average size of 10 nm. b) $CsPbBr_3$ NCs in the fifth day after spin-coated on the substrate with the elemental mappings and EDX spectra.

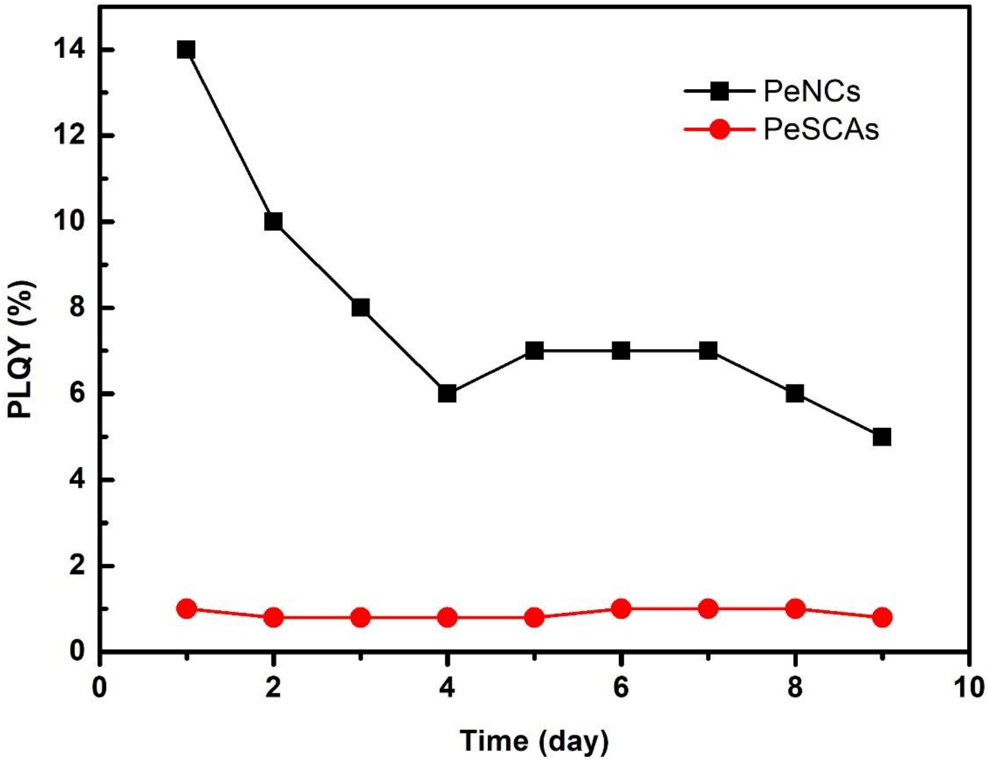


**Figure S12.** PLQY test of PeNCs and PeSCAs within 9 days.

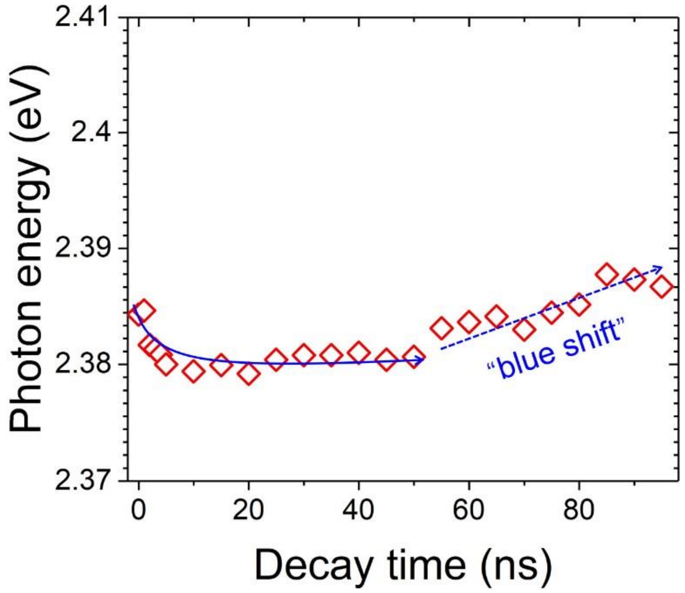


**Figure S13.** Fitted photon energy as a function of decay time for PeSCAs, indicating a "blue-shift" during the decay.

**Table S1.** Elemental distribution from EDX spectrum of $CsPbBr_3$ PeSCAs

| Element | Line Type | Weight % | Weight % Sigma | Atomic % |
|---|---|---|---|---|
| Pb | M series | 38.42 | 1.37 | 21.87 |
| Cs | L series | 21.71 | 1.38 | 19.27 |
| Br | Lseries | 39.87 | 1.14 | 58.86 |
| total | | 100 | | 100 |

**Table S2.** Modeling parameters of horizontal line cuts for PeNCs and PeSCAs

| Sample | Small-structure (nm) | Inter-distance (nm) | Medium structure (nm) | Inter-distance (nm) | Large structure (nm) | Inter-distance (nm) |
|---|---|---|---|---|---|---|
| PeNCs | 4.6 ± 1.4 | 11 ± 4 | 22 ± 8 | 52 ± 20 | 56 ± 13 | 300 ± 75 |
| PeSCAs | 4.9 ± 1.5 | 14 ± 7 | 17 ± 7 | 55 ± 22 | 366 ± 84 | 970 ± 242 |

**Table S3.** Fitting data from the decay curves of PeNCs and PeSCAs.

| Sample | $\tau_1$ (ns) | A1 (%) | $\tau_2$ (ns) | A2 (%) |
|---|---|---|---|---|
| PeNCs | 1.81 ± 0.01 | 13868 | 9.56 ± 0.05 | 4264 |
| PeSCAs | 3.64 ± 0.02 | 5589 | 20.98 ± 0.11 | 2627 |
